# Targeting concerns about falling to modulate biological stress systems: Effects of a multicomponent randomized controlled trial in older adults

**DOI:** 10.1016/j.cpnec.2025.100315

**Published:** 2025-09-01

**Authors:** Anja Müller, Robert Kob, Cornel Christian Sieber, Ellen Freiberger, Nicolas Rohleder, Sabine Britting

**Affiliations:** a Department of Psychology, Chair of Health Psychology, Friedrich-Alexander-Universität Erlangen-Nürnberg, Nägelsbachstrasse 49a, Erlangen, Bavaria, 91058, Germany; b Institute for Biomedicine of Aging, Friedrich-Alexander-Universität Erlangen-Nürnberg, Kobergerstraße 60, Nuremberg, Bavaria, 90408, Germany; c Department of Medicine, Kantonsspital Winterthur, Brauerstrasse 15, Winterthur, 8401, Switzerland

**Keywords:** Concerns about falling, Chronic stress, HPA axis, SNS, Inflammation, Aging, Multicomponent intervention

## Abstract

**Introduction:**

Concerns about falling (CaF) are common in older adults and may act as a chronic stressor affecting physical activity, psychological well-being and physiological regulation. This study examined the impact of a 16-week multimodal exercise intervention on CaF, stress pathways, and peripheral inflammation in older adults.

**Methods:**

In the randomized, controlled FEARFALL study, 160 community-dwelling older adults (aged ≥70 years) were assigned to either an intervention group (IG) or a sham control group (SCG). The IG received a multimodal exercise program, while the SCG engaged in low-intensity activities. Three psychological questionnaires were used to assess CaF: Falls Efficacy Scale-International [FES-I] (fear of falling); Falls Efficacy Scale-International Avoidance Behavior [FES-IAB] (avoidance behavior); Updated Perceived Control of Falling Scale [UP-CoF] (perceived control). Hypothalamic-pituitary-adrenal (HPA) axis and sympathetic nervous system (SNS) activity was determined using saliva samples (cortisol, alpha-amylase), inflammatory markers using blood samples (C-reactive protein [CRP], Interleukin 6 [IL-6]).

**Results:**

There were significant improvements in CaF over time and perceived control in both groups (FES-I: *β* = −6.645, 95 %-CI [-10.56, −2.73], *p* = .001; UP-CoF: *β* = 3.911, 95 %-CI [1.24, 6.58], *p* = .004). Diurnal cortisol slope normalized after the intervention (*β* = −0.014, 95 %-CI [-0.03, 0.00], *p* = .014), while other neuroendocrine and inflammatory markers remained unchanged.

**Conclusion:**

A multimodal short-term intervention reduced psychological aspects of CaF, while physiological stress and inflammatory parameters may require more intensive or longer-term interventions. Findings support CaF as a biopsychosocial stressor and highlight the efficacy of multimodal programs in enhancing coping in older adults.

## Highlights

• Concerns about falling decreased in both groups of older adults.
• Psychological outcomes improved in both groups regardless of intervention type.
• Diurnal cortisol slope improved; other stress and inflammation markers did not.
• Psychological changes emerged quickly; physiological effects may need more time.

## 1 Scientific background

As people grow older, there is an increased risk of falling, which is accompanied by growing *Concerns about Falling* (CaF). These concerns can initiate a negative cycle that includes a decline in *physical activity* (PA), social withdrawal, functional decline and psychological distress. Prolonged stress is associated with dysregulation of stress systems and stimulation of low-grade inflammation [[1], [2], [3], [4]]. PA can protect the body as well as psychological and neuroendocrine systems [[5], [6], [7], [8]]. Multicomponent interventions offer a comprehensive approach to break the cycle of stress, concerns and lack of physical activity in older adults. This study investigates the effects of such interventions on CaF, physiological stress, and peripheral inflammation in older adults.

Despite the common use of the terms *physical activity* and *exercise* as synonyms, the term exercise specifically refers to a structured and planned subset of PA [9]. PA tends to decrease with age due to physiological impairments (e.g., reduced muscle strength, cardiovascular capacity), and psychological factors, including CaF [10]. This decline is associated with an increased risk of falling, a situation that is notably prevalent among the population of older adults [10,11]. Structured multicomponent programs can effectively counteract these declines. They help prevent or delay age-related conditions like sarcopenia [12], reduce fall incidence [13], and improve quality of life in older adults [14]. These programs integrate physical, cognitive, and psychosocial elements, improving physical and cognitive function and reducing anxiety, inflammation, and CaF [[15], [16], [17]].

CaF overlaps significantly with stress-related conditions such as anxiety and may induce physiological effects similar to chronic stress [18]. Chronic stress impacts HPA axis and SNS regulation, which can promote inflammatory activity [1,2]. Dysregulation of stress-related systems, particularly the HPA axis and the SNS, has been linked to inflammatory disinhibition, where altered cortisol dynamics and prolonged SNS activation impair immune regulation, fostering chronic low-grade inflammation [19]. Chronic low-grade inflammation correlates with age-related diseases, including diabetes, frailty, and cardiometabolic disease [20,21]. Elevated C-reactive protein (CRP) and Interleukin 6 (IL-6) levels correlate with increased mortality risk and inversely with successful aging [22,23].

Evidence supports PA's positive effects on anxiety and CaF. Mochcovitch et al. [5] discovered an association between reduced anxiety symptoms and increased PA in older adults. Further research has indicated that PA can reduce CaF experienced by community-dwelling older adults [24,25]. Structured exercise programs improve strength, balance, and confidence in mobility, directly addressing the psychological and physical dimensions of CaF [26,27]. This comprehensive approach emphasizes the dual benefits of PA for mental and physical well-being, enabling older adults to regain independence and improve their quality of life [26,27]. Conversely, CaF can reduce PA levels, creating a harmful cycle [5,28,29], underscoring the need for early interventions to maintain activity in older adults.

Alterations in stress systems could potentially be prevented or counteracted by a number of interventions, notably also by PA: It appears that PA modulates the physiological response to stress by influencing cortisol secretion mediated by the HPA axis. This suggests a potential mechanism through which PA may prevent HPA axis hyperactivation and mitigate the associated physiological and psychological harm [6,30]. The results of a meta-analysis describe the role of PA in optimizing cortisol regulation, suggesting an adaptive response to acute stressors [6]. Moreover, frequent PA has been shown to enhance the balance of autonomic nervous system (ANS) activity by reducing excessive sympathetic activation and promoting parasympathetic function [5].

Research findings indicate that individuals who engage in regular PA exhibit lower concentrations of IL-6 and CRP [31,32]. As a result, regular exercise has been shown to play a key role in reducing systemic inflammation, reducing oxidative damage and improving vascular and muscle strength [7,8]. The particular nature and magnitude of PA executed are also crucial factors. Moderate to high-intensity ranges of PA, like walking and cycling, can alter stress-induced inflammation, while high-intensity workouts can exacerbate inflammatory markers like IL-6 temporarily [33]. A higher level of PA has been associated with lower inflammation and higher functional capacity in older adults [34]. These findings emphasize the central role of PA in improving endocrine resilience to stressors and promoting overall health.

Overall, PA plays a multifaceted role in promoting both mental and physical health in older adults. By alleviating anxiety and reducing concerns about falling, while simultaneously modulating stress-related physiological systems and inflammation, PA contributes to maintaining independence, resilience, and quality of life in later years.

In accordance with previous suppositions that CaF might have similar effects on the body as chronic stress, it can be concluded that stress symptoms in older adults could potentially be relieved by physical activity. Chronic stress can be characterized by decreased HPA axis activity, increased SNS activity, and heightened low-grade inflammation [[1], [2], [3], [4]]. The proposed mechanism by which PA affects the stress axes, low-grade inflammation and fall symptoms is outlined below. Following a multi-intervention program, we hypothesized that (1) SNS activity would decrease and HPA axis activity would increase, (2) inflammatory markers would decrease, and (3) that CaF would decrease. These changes were expected to differ between the intervention group (IG) and a sham control group (SCG).

## 2 Methods

### 2.1 Study design

The data presented in this paper are part of a longitudinal, randomized controlled intervention study that examines the associations between chronic stress and functional health among older adults with CaF (FEARFALL). This project is a collaborative effort between the Institute for Biomedicine of Aging (IBA) and the Chair of Health Psychology at Friedrich-Alexander-Universität Erlangen-Nürnberg. The protocol is pre-registered at Open Science Framework (OSF) (https://doi.org/10.17605/OSF.IO/R4CDX), and the study protocol has been published [35]. The study procedures were approved by the ethics committee of FAU (protocol #317_20 B), and all participants provided written informed consent. All study procedures adhered to the Declaration of Helsinki.

### 2.2 Participants

The present study includes individuals aged 70 years and older, who were invited to participate in a four-month intervention program. Recruitment was conducted using various approaches, including existing contact databases at the IBA, advertisements in local newspapers, flyer distribution, and public transport advertisements. Individuals who expressed interest were subjected to a screening process that incorporated specific inclusion criteria. Inclusion criteria were as follows: age of 70 years or older, a Falls Efficacy Scale-International (FES-I, 16-item) score of ≥20, the ability to independently reach the study center, consent to randomization, and informed consent. Exclusion criteria included a life expectancy of less than 12 months, concurrent participation in another intervention study (physical or medical exercise promotion) at the time of recruitment or within the preceding six months, the use of anti-inflammatory medications (e.g., glucocorticoids, non-steroidal anti-inflammatory drugs [NSAIDs]), or cognitive impairment (MMSE score <24). Participants meeting these criteria were initially assessed through telephone interviews. Those who met the established criteria were subsequently scheduled for an in-person assessment at IBA. Assessments were conducted at three time points: before the start of the intervention program (T0), immediately after the multicomponent intervention (MCI) completion (T1), and eight months post-intervention (T2). The present analyses focus on data from T0 and T1.

A-priori power analyses (G∗Power 3.1.9.2) for the primary outcome (FES-I) assumed a small to medium effect (*f* = 0.15), *α* = .05, power = 0.95, and *r* = 0.50, resulting in a required *N* = 154. Taking into account a dropout rate of 25–30 %, we planned a sample size of *N* = 200. With the actual number achieved of *N* = 160, the sensitivity analysis yielded a power of 80 % for detecting approximately *f* = 0.22 (*d* = 0.45) and a power of 90 % for detecting *f* = 0.26 (*d* = 0.52), which is sufficient to detect small to medium effects.

### 2.3 Measures

In this section, we describe the measures that were used in analyses underlying this manuscript. A comprehensive description of all instruments and measures used in FEARFALL can be found in the published study protocol [35] and preregistration (https://doi.org/10.17605/OSF.IO/R4CDX).

### 2.4 Demographic variables

At baseline, participants were asked to provide a range of demographic data points, including age, gender, body mass index (BMI), and history of previous falls.

#### 2.4.1 Concerns about Falling (CaF)

A comprehensive evaluation of CaF was conducted using three validated questionnaires. To assess fear of falling, we used the validated German version of the Falls Efficacy Scale-International (FES-I), a 16-item instrument designed for community-dwelling older adults [36]. The behavioral consequences of falling were measured with the FES-IAB scale [37], and perceived control over falls was assessed with the Updated Perceived Control over Falling (UP-COF) scale [38].

#### 2.4.2 Stress system activity

Salivary cortisol and salivary alpha-amylase (sAA) diurnal profiles were measured according to established protocols to evaluate basal HPA axis and SNS activity [[39], [40], [41]]. On two consecutive days (D1; D2), participants collected saliva samples using Salivettes (Sarstedt, Nümbrecht, Germany) at home at three different time points: upon waking (S1), 30 min after waking (S2), and a minimum of 14 h after S2, at bedtime (S3). Exclusion was determined by deviations greater than 5 min (S2) or by collection (S3) occurring less than 14 h prior to S2.

Saliva samples were stored at −30 °C until analysis at the laboratory of the Chair of Health Psychology. Samples underwent centrifugation for 5 min at 2000g and 20 °C. The concentration of cortisol was determined using a chemiluminescence immunoassay (CLIA, IBL International GmbH, Hamburg, Germany) in accordance with the manufacturers' instructions. For sAA, an enzyme kinetic assay was employed, as previously outlined in related publications [4]. Each measurement was performed in duplicate, and the intra-assay and inter-assay variation was less than 10 %.

The activity of the basal stress system was quantified using three indices based on the mean of corresponding samples from both days: the cortisol and sAA awakening response (CAR/AAR), defined as the concentration change within 30 min after waking [39], the diurnal slope, and the area under the curve with respect to ground (AUCg), which represents total hormone output.

#### 2.4.3 Systemic inflammation

To assess low-grade inflammation, plasma concentrations of C-reactive protein (CRP) and interleukin 6 (IL-6) were measured. Therefore, blood samples were collected using ethylenediaminetetraacetic acid (EDTA)-coated monovettes (S-Monovette, 2.7 ml, K3E, 1.6 mg EDTA/ml, Sarstedt) with an indwelling venous catheter (Safety-Multifly-Kanüle, Sarstedt). Participants were not required to meet specific criteria, such as fasting. The study physician collected blood samples at T0, T1, and T2 assessment. Immediately after collection, the samples were centrifuged at 2.000 g for 10 min. After centrifugation, plasma was pipetted and frozen at −80 °C. IL-6 and CRP were quantified with high-sensitivity ELISA kits (Quantikine HS, R&D Systems, Minneapolis, MN, USA; CRP High Sensitive ELISA, IBL International GmbH, Hamburg, Germany) with a detection limit of 0.09 pg/mL for IL-6 and 0.044 μg/mL for CRP. Intra- and inter-assay coefficients of variation were reliably below 10 %.

### 2.5 Multicomponent intervention

#### 2.5.1 Intervention Group (IG)

Participants in the IG completed a 16-week multicomponent exercise program. During the first four weeks, they attended two supervised 60-min sessions per week at the IBA. From weeks 5 through 16, they continued to attend one supervised session per week, supplemented by two 30-min sessions at home. The intervention was considered successful if a participation rate of at least 75 % was achieved in the sessions. The program targeted physical function and through a combination of strength, balance, gait, and functional training, along with cognitive-behavioral strategies adapted from the Matter of Balance program [42]. Strength training followed the Otago protocol using adjustable ankle weights. Balance and gait components included progressive and task-specific exercises, functional training focused on sit-to-stand transitions and stability. The CaF training addressed goal setting, avoidance reduction, and self-efficacy. Home exercise materials were provided to support ongoing engagement.

#### 2.5.2 Sham Control Group (SCG)

The SCG followed the same 16-week schedule. After a 40-min introductory health lecture, each session included 20 min of light upper-body stretching. The groups consisted of 20–30 participants. The program combined light PA with health education. Participants received printed materials on lecture topics and stretching exercises for home use.

#### 2.5.3 Randomization

Randomization was conducted using the online tool Sealed Envelope (Sealed Envelope Ltd., London, UK) and was performed by a separate member of the intervention team. To ensure double blinding, the intervention and evaluation tasks were executed by different teams. The evaluation team, which was responsible for outcome assessment and data management, had no access to participants' group assignments and was independent from the intervention team. In order to guarantee comparable group distribution, stratification was based on gender and statin use. Couples were block-randomized to allow joint participation. After randomization, participants received a letter with general information and the scheduled dates for the individual intervention appointments.

### 2.6 Statistics

All statistical analyses were conducted using R version 4.2.2, with the dplyr, lme4, nlme, MuMIn, and stats packages utilized for model estimation. Visualization was carried out using ggplot2 and sjPlot. In order to address the issue of skewness and normalize the data, raw values of cortisol, IL-6 and CRP concentrations were log-transformed (x log = log10 (x + 1)), sAA was sqrt-transformed (x sqrt = SQRT (x + 1)), and adherence tests were performed.

Linear Mixed Models (LMMs) were used for the statistical analysis of repeatedly measured CaF, stress-related and inflammatory parameters. The sum scores of FES-I, FES-IAB and UP-CoF were used as dependent variables for CaF, cortisol and sAA parameters (CAR/AAR, slope, AUCg) for basal stress system activity, and the concentrations of IL-6 and CRP as markers for inflammation. The models included the fixed effects of group (between-subject), time (within-subject) and their interaction (“group × time”) to assess potential intervention effects. To control for potential confounding (FES-I, FES-IAB, UP-CoF), Age, Sex and BMI were included as covariates. Random intercepts at subject level (SubID) were modeled to account for individual variability in repeated measures.

Contrary to our study protocol in which we originally aimed to include only participants with an adherence >75 % to the intervention, we decided to include data from all participants in the analysis, to avoid an additional data loss of about 20 %. We have conducted additional analyses using the sample with >75 % adherence and report any results that differ using this smaller data set.

All tests were two-tailed with a significance level of *p* < .05. The results of the fixed effects are reported as estimated regression coefficients (*β*) with standard errors and 95 % confidence intervals. Model performance was evaluated using AIC values and (optionally) R^2^ statistics if applicable.

## 3 Results

### 3.1 Characteristics of the study sample

**Fig. 1:**
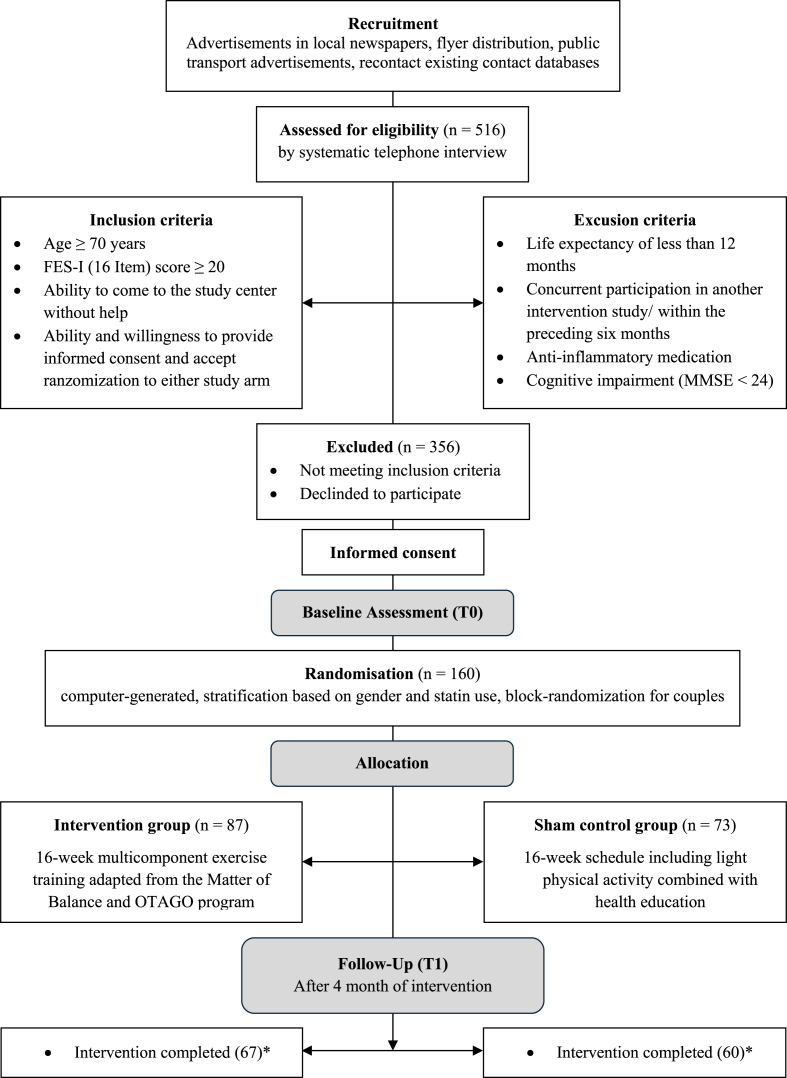
Flow of participants through the trial. Note: ∗The intervention was considered completed if 75 % participation was achieved.

Table 1 presents the baseline characteristics of the participants in the IG (n = 87) and the SCG (n = 73) (see Fig. 1 for the participant flow through the study). On average, the groups were of a similar age, with a comparable proportion of women and men and a similar body mass index. Additionally, no substantial disparities were observed in other characteristics, including the proportion of individuals residing alone, the frequency of falls, and the occurrence of injury-related falls. The psychometric values (FES-I, FES-IAB, UP-CoF, PSS-10, GAS) were comparable between the two groups. The inflammation markers C-reactive protein (CRP) and interleukin-6 (IL-6) exhibited only minor variations. A comparison of the groups at T0 revealed comparable initial values.

**Table 1:** Baseline sample characteristics.

	IG		SCG		*p*
	(n = 87)		(n = 73)		
	n/mean	%/SD	n/mean	%/SD	
Age (years)	79.83	5.59	79.16	4.92	0.431
Women	64	73.60	54	74.00	0.410
Men	23	26.40	19	26.00	0.639
BMI [kg/m^2^]	26.31	4.47	26.91	3.97	0.386
Living alone	51	58.60	37	50.70	0.318
Falls [n, %]	58	66.70	44	60.30	0.915
injurious falls [n, %]	36	41.40	31	42.50	0.891
FES-I	30.36	8.29	29.79	7.81	0.662
FES-IAB	25.03	7.45	24.77	8.01	0.827
UP-CoF n = 71 (IG); n = 67 (SCG)	13.77	4.32	13.10	3.97	0.345
PSS-10	24.94	6.88	24.63	6.80	0.774
GAS	13.69	7.44	15.32	8.30	0.194
CRP [μg/ml] n = 79 (IG); n = 67 (SCG)	1.67	1.94	1.37	1.29	0.471
IL-6 [pg/ml] n = 80 (IG); n = 67 (SCG)	2.25	1.50	2.31	1.62	0.601

Note: Intervention Group (IG), Sham Control Group (SCG), mean (M), standard deviation (SD), p-value (p): p-values are based on independent samples t-tests, body mass index (BMI), Falls Efficacy Scale-International (FES-I); FES-I total score ranging from 16 to 64. 16–19 low, 20–27 moderate, 28–64 high [36], Falls Efficacy Scale-International Avoidance Behavior (FES-IAB); FES-IAB score ranging from 16 to 64; higher scores represent higher avoidance behavior [37], Updated perceived model of Falling Scale (UP-CoF); UP-CoF score ranging from 0 to 20. A Score lower 13 indicates low perceived control [38], Perceived Stress Scale 10 (PSS-10); PSS-10 score ranging from 10 to 50, higher scores represent higher levels of stress [49], Geriatric Anxiety Scale (GAS); GAS total score ranging from 0 to 54; 1–11 minimal, 12–21 mild, 22–27 moderate, 28–54 severe [50], C-reactive protein (CRP), Interleukin 6 (IL-6).

### 3.2 Intervention effects

Linear mixed models were calculated to examine the change caused by the intervention. The subsequent results are presented separately to address CaF, basal HPA axis activity, basal SNS activity, and inflammatory processes.

#### 3.2.1 Concerns about falling

**Fig. 2:**
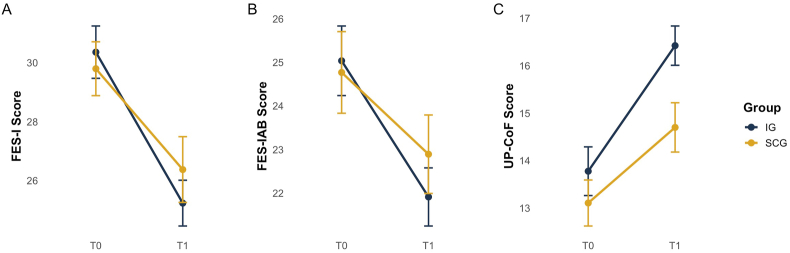
Changes in CaF over time (T0 to T1) for Intervention and Sham Control Group. Note: Falls Efficacy Scale-International (FES-I); Falls Efficacy Scale-International Avoidance Behavior (FES-IAB); Updated perceived model of Falling Scale (UP-CoF); Intervention Group (IG), Sham Control Group (SCG); pre-Intervention (T0); post-Intervention (T1).

There was a significant main effect of time on FES-I (*β* = −6.645, 95 %-CI [−10.56, −2.19], *p* = .001), indicating a decrease in CaF over time. The FES-IAB showed a decline on a descriptive level, but changes failed to reach statistical significance (*β* = −3.439, 95 %-CI [−7.18, 0.30], *p* = .071). The UP-CoF exhibited a significant positive time effect (*β* = 3.911, 95 %-CI [1.24, 6.58], *p* = .004), suggesting an enhancement in perceived control over falls. No significant interaction between time and group was identified for any of the three measures (FES-I: *β* = 1.573, 95 %-CI [−0.95, 4.10], *p* = .221; FES-IAB: *β* = 0.876, 95 %-CI [−1.54, 3.29], *p* = .475; UP-CoF: *β* = −1.131, 95 %-CI [−2.83, 0.57], *p* = .190). This indicates that changes over time were detected for FES-I and UP-CoF, but there was no difference in the respective changes between groups (Fig. 2).

Separate analyses using the sample with >75 % adherence to the intervention did not change the majority of the results. While the analysis of the larger sample showed only a trend towards a decline in avoidance behavior (FES-IAB) over time, the analysis of the smaller sample (with 75 % participation in the intervention) was significant (*β* = −5.05, 95 %-CI [−9.005, −1.10], *p* = .012).

In the models for FES-I and FES-IAB, the covariates age and BMI exhibited a significant positive association with the respective CaF measures (age: *β* = 0.416, 95 %-CI [0.20, 0.63], *p* < .001; *β* = 0.450, 95 %-CI [0.27, 0.63], *p* < .001; BMI: *β* = 0.308, 95 %-CI [0.05, 0.57], *p* = .020; *β* = 0.324, 95 %-CI [0.10, 0.54], *p* = .004). The effects of sex FES-IAB were statistically significant (*β* = −2.667, 95 %-CI [−4.84, −0.50], *p* = .016). Specifically, female participants reported higher scores compared to their male counterparts. No covariates were found to be significant for UP-CoF (all p > .05) (Table 2).

**Table 2:** Results of statistical analyses of CaF over time (T0 to T1) for Intervention and Sham Control Group.

Predictors	FES-I (n = 151)			FES-IAB (n = 151)			UP-CoF (n = 150)		
	Estimates	CI	p	Estimates	CI	p	Estimates	CI	p
Group	−1.971	−6.13 – 2.19	0.352	−0.643	−4.51 – 3.22	0.743	0.522	−2.18 – 3.23	0.704
Time	−6.645	−10.56–−2.73	**.001**	−3.439	−7.18 – 0.30	0.071	3.911	1.24–6.58	**.004**
Group × Time	1.573	−0.95 – 4.10	0.221	0.876	−1.54 – 3.29	0.475	−1.131	−2.83 – 0.57	0.190
Age	0.416	0.20–0.63	**.001**	0.450	0.27–0.63	**<.001**	−0.093	−0.20 – 0.01	0.091
Sex	−2.552	−5.12 – 0.02	0.052	−2.667	−4.84–−0.50	**.016**	0.266	−1.04 – 1.58	0.689
BMI	0.308	0.05–0.57	**.020**	0.324	0.10–0.54	**.004**	−0.093	−0.23 – 0.04	0.167
**Random Effects**									
ICC	0.58			0.48			0.36		
R^2^ (marginal/conditional)	0.171/0.652			0.176/0.571			0.118/0.432		

Note: Falls Efficacy Scale–International (FES-I); Falls Efficacy Scale–International Avoidance Behavior (FES-IAB); Updated Perceived Control over Falling Scale (UP-CoF), body mass index (BMI), intra-class correlation coefficient (ICC).

#### 3.2.2 Basal HPA axis activity

**Fig. 3:**
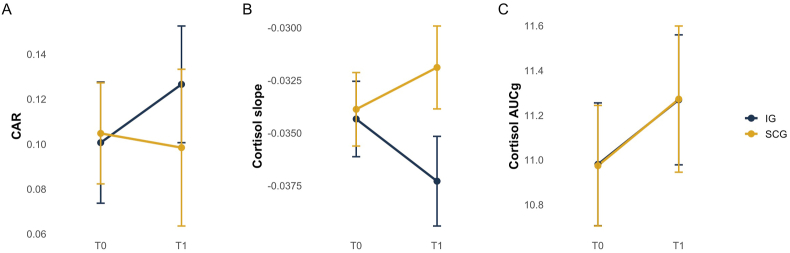
Changes in cortisol indices over time (T0 to T1) for Intervention and Sham Control Group. Note: Cortisol awakening response (CAR); Cortisol Area under the Curve with respect to the ground (Cortisol AUCg); Intervention Group (IG), Sham Control Group (SCG); pre-Intervention (T0); post-Intervention (T1).

Basal HPA axis activity was described by CAR, cortisol slope, and the AUCg (Fig. 3). There were no significant group differences, nor any changes over time, for CAR and AUCg. A statistically significant negative time effect was demonstrated for cortisol slope (*β* = −0.014, 95 %-CI [−0.03, −0.00], p = .014), yet no main effect of the group was observed (*β* = −0.010, 95 %-CI [−0.02, 0.00], *p* = .082). The interaction between group and time exhibited trend-level in cortisol slope that nearly reached the conventional significance level (*β* = 0.007, 95 %-CI [−0.00, 0.24], *p* = .051). Further, higher BMI was associated with a flatter cortisol diurnal curve (*β* = 0.001, 95 %-CI [0.00, 0.01], *p* = .023). The remaining covariates demonstrated no significant influence (*p* > .05) (Table 3).

**Table 3:** Results of statistical analyses of Cortisol and sAA indices over time (T0 to T1) for Intervention and Sham Control Group.

*Predictors*	HPA-Axis								
	CAR (N = 120)			Slope (N = 120)			AUCg (N = 116)		
	*Estimates*	*CI*	*p*	*Estimates*	*CI*	*p*	*Estimates*	*CI*	*p*
Group	−0.075	−0.25 – 0.10	0.398	−0.010	−0.02 – 0.00	0.082	−0.619	−1.93 – 0.69	0.351
Time	−0.063	−0.24 – 0.11	0.483	−0.014	−0.03 – 0.00	**.014**	−0.445	−0.04 – 0.06	0.767
Group × Time	0.055	−0.06 – 0.17	0.342	0.007	−0.00 – 0.01	0.051	0.468	−0.27 – 1.21	0.213
FES-I	−0.002	−0.01 – 0.00	0.484	−0.000	−0.00 – 0.00	0.770	0.007	−0.04 – 0.06	0.767
FES-IAB	0.001	−0.01 – 0.01	0.875	−0.000	−0.00 – 0.00	0.093	−0.003	−0.06 – 0.05	0.914
UP-CoF	0.001	−0.01 – 0.01	0.713	−0.000	−0.00 – 0.00	0.529	0.043	−0.03 – 0.11	0.219
PSS-10	−0.002	−0.01 – 0.00	0.371	−0.000	−0.00 – 0.00	0.178	0.025	−0.03 – 0.08	0.322
Age	−0.001	−0.01–0.01	0.717	0.000	−0.00 – 0.00	0.105	0.035	−0.05 – 0.12	0.397
Sex	−0.035	−0.11 – 0.04	0.341	−0.004	−0.01 – 0.00	0.199	−0.397	−1.33 – 0.53	0.400
BMI	−0.002	−0.01 – 0.01	0.630	0.001	0.00–0.00	**.023**	0.067	−0.03 – 0.16	0.167
**Random Effects**									
ICC	0.09			0.41			0.77		
R^2^ (marginal/conditional)	0.033/0.123			0.098/0.468			0.038/0.778		

*Predictors*	SNS								
	AAR (N = 120)			Slope (N = 121)			AUCg (N = 12β)		
	*Estimates*	*CI*	*p*	*Estimates*	*CI*	*p*	*Estimates*	*CI*	*p*
Group	0.405	−1.70 – 2.51	0.705	−0.002	−0.15 – 0.15	0.980	5.363	−22.65 – 33.38	0.706
Time	0.362	−1.75 – 2.47	0.736	−0.003	−0.15 – 0.15	0.972	5.850	−19.34 – 31.04	0.647
Group × Time	−0.094	−1.45 – 1.26	0.891	0.003	−0.09 – 0.10	0.958	−3.226	−19.11 – 12.66	0.689
FES-I	0.008	−0.06 – 0.08	0.822	−0.003	−0.01 – 0.00	0.263	−0.494	−1.56 – 0.57	0.362
FES-IAB	−0.021	−0.10 – 0.06	0.609	0.002	−0.00 – 0.01	0.447	0.358	−0.82 – 1.54	0.550
UP-CoF	−0.097	−0.20 – 0.01	0.064	−0.005	−0.01 – 0.00	0.225	−1.849	−3.33–−0.37	**.015**
PSS-10	−0.066	−0.13–−0.00	**.044**	−0.005	−0.01–0.00	**.024**	0.043	−1.02 – 1.10	0.936
Age	−0.033	−0.12 – 0.05	0.446	−0.004	−0.01 – 0.00	0.231	0.279	−1.40 – 1.96	0.743
Sex	1.102	0.12–2.08	**.028**	0.040	−0.03 – 0.11	0.283	1.427	−18.17 – 21.03	0.886
BMI	−0.022	−0.12 – 0.08	0.661	−0.002	−0.01 – 0.01	0.581	0.551	−1.43 – 2.53	0.584
**Random Effects**									
ICC	0.29			0.35			0.75		
R^2^ (marginal/conditional)	0.070/0.339			0.054/0.390			0.031/−0.763		

Note: Cortisol awakening response (CAR), alpha-amylase awakening response (AAR); area under the curve with respect to the ground (AUCg); Falls Efficacy Scale–International (FES-I); Falls Efficacy Scale–International Avoidance Behavior (FES-IAB); Updated Perceived Control over Falling Scale (UP-CoF); Perceived Stress Scale 10 (PSS-10); Body-Mass-Index (BMI); intra-class correlation coefficient (ICC).

Separate analyses using the sample with >75 % adherence to the intervention did not change the majority of the results. However, there was a significant effect for FES-IAB on cortisol slope (*β* = −0.001, 95 %-CI [−0.001, −0.000], *p* = .049), indicating that a higher FES-IAB was associated with a flatter slope.

#### 3.2.3 Basal SNS activity

Basal SNS activity was described analogously to the cortisol parameters by AAR, sAA slope, and AUCg. No significant group differences or changes over time were detected for any of the three parameters.

A significant covariate effect was identified in the model for AAR: male participants demonstrated a more pronounced sAA awakening response compared to female participants (*β* = 1.102, 95 %-CI [0.12, 2.08], *p* = .028). Moreover, a higher PSS score was associated with a flatter AAR and a steeper sAA slope (*β* = −0.066, 95 %-CI [−0.13, −0.00], *p* = .044; *β* = −0.005, 95 %-CI [−0.01, −0.00], *p* = .024). Furthermore, participants who reported a higher perceived control exhibited lower overall cortisol levels (*β* = −1.849, 95 %-CI [−3.33, 0.37], *p* = .015) (Table 3).

Separate analyses using the sample with >75 % adherence to the intervention did not change the majority of the results. While the analysis of the larger sample showed only a trend towards higher perceived control with a flatter AAR, the analysis of the smaller sample (with 75 % participation in the intervention) was significant (*β* = −0.111, 95 %-CI [−0.22, −0.00], *p* = .046).

#### 3.2.4 Inflammatory markers

The levels of the inflammatory markers IL-6 and CRP were analyzed to assess the presence of low-grade inflammatory processes (Table 4). The analysis revealed no significant changes over time or group differences. A significant covariate effect was identified for IL-6 with age (*β* = 0.049, 95 %-CI [0.04, 0.32], *p* = .011) and BMI (*β* = 0.097, 95 %-CI [0.05, 0.14], *p* < .001). IL-6 levels increased with age and higher BMI. BMI demonstrated a significant positive association with higher CRP level (*β* = 0.086, 95 %-CI [0.02, 0.15], *p* = .010).

**Table 4:** Results of statistical analyses of inflammatory markers over time (T0 to T1) for Intervention and Sham Control Group.

Predictors	IL-6 (N = 142)			CRP (N = 141)		
	Estimates	CI	p	Estimates	CI	p
Group	0.209	−0.87 – 1.29	0.704	−0.511	−1.96 – 0.94	0.487
Time	0.132	−0.97 – 1.24	0.814	0.104	−1.37 – 1.58	0.890
Group × Time	−0.180	−0.88 – 0.52	0.613	0.134	−0.79 – 1.06	0.776
FES-I	0.013	−0.02 – 0.05	0.462	0.022	−0.03 – 0.07	0.366
FES-IAB	0.008	−0.03 – 0.05	0.689	−0.017	−0.07 – 0.04	0.541
UP-CoF	−0.003	−0.05 – 0.05	0.903	−0.024	−0.10 – 0.05	0.518
PSS	−0.004	−0.03 – 0.03	0.791	−0.028	−0.07 – 0.02	0.202
Age	0.049	0.01–0.09	**.011**	−0.015	−0.07 – 0.04	0.610
Sex	0.052	−0.37 – 0.48	0.810	−0.078	−0.72 – 0.56	0.809
BMI	0.097	0.05–0.14	**<.001**	0.086	0.02–0.15	**.010**
**Random Effects**						
ICC	0.03			0.22		
R^2^ (marginal/conditional)	0.154/0.181			0.054/0.260		

Note: Interleukin 6 (IL-6); C-reactive protein (CRP); Falls Efficacy Scale–International (FES-I); Falls Efficacy Scale–International Avoidance Behavior (FES-IAB); Updated Perceived Control over Falling Scale (UP-CoF); Perceived Stress Scale 10 (PSS-10); Body-Mass-Index (BMI); intra-class correlation coefficient (ICC).

Separate analyses using the sample with >75 % adherence to the intervention did not change the results.

## 4 Discussion

The purpose of this study was to examine the impact of a multicomponent intervention on CaF, physiological stress markers (cortisol, sAA), and inflammatory parameters (IL-6, CRP) in community-dwelling older adults aged 70 and older. In line with our hypotheses, we observed a significant decrease in concerns about falling (FES-I) and an increase in perceived control over falls (UP-CoF) over time, but in contrast to our hypotheses, these changes did not differ between intervention and control groups. Although some changes in physiological parameters occurred after the intervention, particularly a significant change in individual cortisol levels, other cortisol indices, sAA indices and inflammatory markers remained largely unchanged.

Although the analysis of the adherent sample strictly complies with the study protocol, it is beneficial to report and discuss the results of the larger overall sample in detail. Firstly, there are no substantial differences between the two analyses, which highlights the robustness of the findings. On the other hand, the adherent sample confirms the main results more clearly in some cases, with marginally significant effects in the overall sample achieving statistical significance in the adherent sample.

The results regarding the reduction in CaF are consistent with existing recommendations and research findings on the effects of PA and multicomponent interventions in older adults [24,25]. Regarding CaF, participants demonstrated a significant decrease over time (FES-I), alongside an increase in their perceived control (UP-CoF). These outcomes align with previous studies emphasizing the beneficial effects of PA on psychological well-being in older adults, including reduced anxiety and improved confidence in balance and mobility [5,24,25,28,29]. It appears that, in particular, CaF manifests as a psychological variable, demonstrating robust psychological effects. One potential explanation for this outcome is that all participants received an equivalent level of attention and engagement in terms of communication and social interaction. Such interactions, along with regular reflection on health status, have been identified as contributing factors to the significant improvements observed. The role of social interaction and repeated functional assessments (e.g., mobility tests) may have positively influenced CaF. Similar findings have been reported in previous studies [43].

In this study, CaF are conceptualized as a chronic stressor. It has been demonstrated that persistent stress, such as concerns (about falling), can be associated with dysregulation of stress systems and low-grade inflammation [[1], [2], [3], [4]]. Physical activity (PA) is considered a protective factor, as it can modulate stress systems such as the HPA axis and SNS, and is theoretically associated with improved regulation of physiological stress responses [[5], [6], [7], [8]]. However, the present study did not provide clear evidence of corresponding changes in biological stress or inflammation markers. The findings suggest that the hypothesized physiological effects were not detectable under the given conditions, possibly due to the limited duration and intensity of the intervention. It is also conceivable that such effects require longer periods to emerge.

Nevertheless, the findings regarding biological stress markers were less conclusive. Although a modest improvement in the diurnal cortisol slope was observed, suggesting some normalization in HPA axis activity, other parameters, such as CAR, AUCg, and sAA analyses, remained unchanged. In addition, no significant group differences were observed. One explanation could be that the intervention's duration, intensity, or frequency was insufficient to elicit measurable biological adaptations. This interpretation aligns with previous studies reporting similar observations [6].

Inflammatory markers (IL-6, CRP) showed no significant changes. This contradicts expectations and some prior research suggesting an anti-inflammatory effect of regular exercise [7,8,34]. However, significant associations with age and BMI were found, consistent with earlier findings [20]. This supports the notion that chronic low-grade inflammation is more likely to be modifiable through sustained lifestyle changes rather than short-term interventions. Furthermore, previous cross-sectional analyses of the FEARFALL study revealed no significant correlations between CaF, basal stress system activity, and inflammatory markers.

In contrast, the lack of changes in physiological parameters is not unexpected in populations aged 70 and older. It is known that neuroendocrine responses become less adaptive with age. Neuroendocrine plasticity declines with age, the HPA axis becomes less flexible, SNS activity often remains elevated, and inflammaging is difficult to modulate through moderate PA [44,45]. Additionally, biomarkers such as cortisol, IL-6, and CRP are known to respond slowly to interventions and are influenced by external factors. A 12-week intervention might therefore be too brief to induce measurable effects [31].

Moreover, studies involving voluntary participants often exhibit a “healthy participant bias”, in this case implying that participants tend to be healthier and more active than the general population, which may lead to improvements in both groups [46,47]. It is also important to recognize that psychological responses (e.g., reduced anxiety) typically manifest more rapidly than physiological responses, which require longer durations and higher intensities of stimulation.

The findings suggest that the intervention primarily yielded psychological benefits, with limited evidence for short-term physiological change. Future research is warranted to explore physiological changes in this age group, which may require longer-term, higher-intensity, or more targeted interventions.

### 4.1 Limitations

Despite the promising psychological outcomes, several limitations should be considered. The intervention's duration may have been insufficient to elicit biological changes, which often require prolonged exposure. The regular, positive interaction with the study staff may have functioned as a social reinforcer, thereby inducing a learning psychology effect. Analogous to the Hawthorne effect, this could explain why the SCG also exhibited comparable developments in relation to the CaF as the intervention group [48].

As discussed above, the sample as included relatively healthy, motivated individuals, limiting generalizability to frailer older adults (“healthy participant bias”) [46]. This suggests that a selective presentation of results is inappropriate and that reporting the larger sample in detail is a conservative approach that strengthens the generalizability of the findings.

Lastly, biological responses might not have been observable immediately post-intervention due to potential delayed effects. In addition, the assessment of salivary cortisol and alpha-amylase entails methodological constraints. These include pronounced diurnal variability, acute sensitivity to stressors, and high inter-individual baseline variability. All of these factors may limit the interpretability of the results.

### 4.2 Strengths of the study

To comprehensively map the activity of the stress axis, specific indices were calculated in addition to the cortisol and sAA values measured individually, such as the awakening curve, diurnal slope, and area under the curve (AUCg). This ensured that stress axis activity was recorded from several different perspectives.

The assessment of CaF encompassed multiple dimensions. Rather than using a single questionnaire for assessment, various psychological constructs were considered, including fear of falling, avoidance behavior, and perceived control. The aim was to capture the different facets of fear of falling as broadly and specifically as possible.

It should be emphasized that the number of contacts in the SCG was comparable to that in the IG. In both groups, the regular training sessions promoted physical activity, social participation, mutual exchange, and psychological well-being. This approach is methodologically interesting and ethically relevant, as all participants benefited from the study.

### 4.3 Implications for future research

The results underscore the intervention's effectiveness in improving psychological outcomes, especially CaF. Increasing the intensity or duration of the intervention may help to reveal physiological effects. Additionally, long-term follow-up assessments could provide clearer insights into the temporal dynamics of biological responses. Particular attention should be paid to group dynamics, which appear to play a key role in modifying CaF. This may help to interrupt the cycle of fear, avoidance, and inactivity, and strengthen self-efficacy in older adults.

## 5 Conclusion

This study demonstrates that a multicomponent intervention, as well as a sham intervention, can significantly reduce CaF and enhance perceived control in community-dwelling older adults. While physiological stress and inflammatory markers remained largely unchanged immediately after intervention, the clear psychological improvements highlight the potential of low-threshold interventions to positively influence emotional well-being and confidence in daily mobility. These findings support the integration of structured, group-based PA programs into routine geriatric care as a non-pharmacological means to strengthen self-efficacy and prevent inactivity-related health decline. Future programs should emphasize social engagement and continuity to maximize psychological benefits and potentially foster longer-term physiological adaptation.

## CRediT authorship contribution statement

**Anja Müller:** Writing – original draft, Visualization, Investigation, Formal analysis, Data curation, Conceptualization. **Robert Kob:** Writing – review & editing, Methodology, Conceptualization. **Cornel Christian Sieber:** Resources. **Ellen Freiberger:** Writing – review & editing. **Nicolas Rohleder:** Writing – review & editing, Supervision, Methodology, Formal analysis, Conceptualization. **Sabine Britting:** Writing – review & editing, Supervision, Project administration, Methodology, Investigation, Data curation, Conceptualization.

## Data availability

The datasets generated and analyzed as part of the current study are not publicly available, but can be requested from the corresponding author.

## Ethics statement and privacy rights

The study protocol was approved by ethics committee and complies with the Declaration of Helsinki and Guidelines for Good Clinical Practice. Ethics approval was obtained by local IRB (Ethikkommission der Medizinischen Fakultät der FAU (Friedrich-Alexander Universität Erlangen-Nürnberg)), Germany, #317_20B, August 26, 2020. This study was registered at German Clinical Trials Register (identifier: DRKS00029171). All data protection regulations were strictly followed. At the baseline visit, all participants have to sign written, informed consent forms prior to assessments and receive a written information sheet containing the most relevant study components. Changes to the protocol will be approved by the ethical committee are reported to German Clinical Trials Register. An insurance is provided to all participants for all assessments and the intervention during the period of study participation.

## Declaration of generative AI and AI-assisted technologies in the writing process

During the preparation of this work, the authors used ChatGPT (GPT‐4o; OpenAI, San Francisco, CA, USA) in order to enhance readability and optimize code in R. After using this tool/service, the authors reviewed and edited the content as needed and take full responsibility for the content of the publication.

## Funding

FEARFALL is funded by the 10.13039/501100001659Deutsche Forschungsgemeinschaft (10.13039/501100001659DFG, 10.13039/501100001659German Research Foundation) [465677295].

## Declaration of competing interest

The authors declare that they have no known competing financial interests or personal relationships that could have appeared to influence the work reported in this paper.

## Glossary

BIA: Bioelectrical impedance analysis
BMI: Body Mass Index
CaF: Concerns about falling
CAR: Cortisol awakening response
CRP: C-reactive protein
FES-I: Falls Efficacy Scale International
FES-IAB: FES-I Avoidance Behavior
FoF: Fear of falling
GAS: Geriatric Anxiety Scale
GDS: Geriatric Depression Scale
HPA: Hypothalamus-pituitary-adrenal
IBA: Institute for Biomedicine of Aging
IG: Intervention group
IL-6: Interleukin 6
PSS: Perceived Stress Scale
sAA: Salivary alpha-amylase
SCG: Sham control group
SNS: Sympathetic nervous system
UP-COF: Updated Perceived Control over Falling Scale
